# Anticonvulsive Effects of Chondroitin Sulfate on Pilocarpine and Pentylenetetrazole Induced Epileptogenesis in Mice

**DOI:** 10.3390/molecules26226773

**Published:** 2021-11-09

**Authors:** Shareen Singh, Thakur Gurjeet Singh, Manjinder Singh, Agnieszka Najda, Renata Nurzyńska-Wierdak, Rafa Almeer, Mohamed Kamel, Mohamed M. Abdel-Daim

**Affiliations:** 1 Chitkara College of Pharmacy, Chitkara University, Changdigarh 140401, Punjab, India; shareen.singh@chitkara.edu.in (S.S.); manjinder.singh@chitkara.edu.in (M.S.); 2 Department of Vegetable Crops and Medicinal Plants, University of Life Sciences in Lublin, 50A Doświadczalna Street, 20-280 Lublin, Poland; agnieszka.najda@up.lublin.pl (A.N.); renata.nurzynska@up.lublin.pl (R.N.-W.); 3 Department of Zoology, College of Science, King Saud University, P.O. Box 2455, Riyadh 11451, Saudi Arabia; ralmeer@ksu.edu.sa; 4 Department of Medicine and Infectious Diseases, Faculty of Veterinary Medicine, Cairo University, Giza 12211, Egypt; m_salah@cu.edu.eg; 5 Pharmacology Department, Faculty of Veterinary Medicine, Suez Canal University, Ismailia 41522, Egypt

**Keywords:** chondroitin sulfate, extracellular matrix, intracellular ionic concentration, caspase-3, pro-inflammatory mediators, oxidative stress

## Abstract

Chondroitin sulfate is a proteoglycan component of the extracellular matrix (ECM) that supports neuronal and non-neuronal cell activity, provides a negative domain to the extracellular matrix, regulates the intracellular positive ion concentration, and maintains the hypersynchronous epileptiform activity. Therefore, the present study hypothesized an antiepileptic potential of chondroitin sulfate (CS) in pentylenetetrazole-induced kindled epilepsy and pilocarpine-induced status epilepticus in mice. Levels of various oxidative stress markers and inflammatory mediators were estimated in the brain tissue homogenate of mice, and histopathological changes were evaluated. Treatment with valproate (110 mg/kg; i.p.) as a standard drug and chondroitin sulfate (100 & 200 mg/kg, p.o.) significantly (*p* < 0.01) and dose-dependently prevented the severity of kindled and spontaneous recurrent seizures in mice. Additionally, chondroitin sulfate showed its antioxidant potential by restoring the various biochemical levels and anti-inflammatory properties by reducing NF-kB levels and pro-inflammatory mediators like TNF-alpha, IL-1β, and IL-6, indicating the neuroprotective effect as well as the suppressed levels of caspase-3, which indicated a neuroprotective treatment strategy in epilepsy. The proteoglycan chondroitin sulfate restores the normal physiology and configuration of the neuronal tissue. Further, the molecular docking of chondroitin sulfate at the active pockets of TNF-alpha, IL-1β, and IL-6 showed excellent interactions with critical amino acid residues. In conclusion, the present work provides preclinical evidence of chondroitin sulfate as a new therapeutic approach in attenuating and preventing seizures with a better understanding of the mechanism of alteration in ECM changes influencing abnormal neuronal activities.

## 1. Introduction

Epilepsy is defined as the second most common neurological condition characterized by sudden recurrent episodes of epileptic seizures unprovoked by any immediately identifiable cause and is manifested clinically by abnormal behavior changes due to aberrant electrical discharges of set neurons in the brain [1]. As per the WHO report of 2021, approximately 50 million people are suffering from epilepsy worldwide, out of which 80% of epileptic persons belong to low- and middle-income countries. An epileptic seizure is the clinical manifestation of an abnormal and excessive electrical discharge of a set of neurons in the brain [2]. The currently available pharmacotherapy for epilepsy is not permanently targeted in curing the disease but is effective as a symptomatic treatment for different types of seizures by suppressing the occurrence time of these seizures, which are recognized as the symptomatology of epilepsy. Thus, the available antiepileptic drugs in many patients are quite successful in preventing the recurrence of these seizures, and they have been shown to provide a remarkable improvement in the clinical condition of some patients. As per the clinical reports, one-third of patients gets refractory seizures when using these medications [3]. However, none of the available drugs conclusively addresses the problem of containing the progressive increase in seizure severity, a process by which a near-normal brain turns epileptic.

The mechanistic approaches of these available antiepileptic drugs targeting various pathways have been elaborated by pharmacological evidence in considerable detail, including some of the important molecular signaling mechanisms underlying epileptogenesis. Enhanced electrical activity in the epileptic brain has been observed with alterations in the extracellular space composed of extracellular matrix and extracellular fluid modulating ionic concentration [2,4]. Extracellular space volume comprises the interstitial fluid and the extracellular matrix; the interstitial fluid is essential for determining the ionic concentration (Ca^2+^, Cl^−^, Mg^2+^, and Na^+^), neurotransmitters, and electric field interaction between neurons [4]. The other extracellular matrix supporting the neighboring cells comprises many proteoglycans (hyaluronan, lecticans, tenascins, heparin sulfate, and chondroitin sulfate) [5]. The alterations in the volume fraction (α) of the brain’s extracellular space (ECS) can prolong or even initiate seizures. In epilepsy, the observed shrinkage of the brain causes the decrease in the extracellular space leading to the shift of ionic concentration between the intracellular region and the extracellular region by increasing the intracellular space of ion concentration as compared to the extracellular region of the neuronal cell [5,6]. The alterations of extracellular space (ECS), i.e., the decreased extracellular space (ECS), lead to the increase in the ephaptic interactions between excitatory neurotransmitter signaling, causing the excitability of the neurons [7]. Alterations in the interstitial fluid comprised of ions (Ca^2+^, Cl^−^, Mg^2+^, and Na^+^) allowing the movement of electric field interaction between neurons by release of neurotransmitters is affected, which might be a reason why the increased intracellular positive ionic concentration has been hypothesized to increase the excitability in epilepsy [5,6]. The other changes in the extracellular matrix part that make up the ECS, including proteoglycan, support the neuronal and non-neuronal cells, which are determined by the decreased levels of proteoglycans modulating the neuronal activities, which might be a reason for decreased GABA-ergic and increased glutamatergic neurotransmission [8,9].

The study hypothesized that proteoglycan chondroitin sulfate provides a negative domain to the extracellular matrix and regulates the extracellular positive ion concentration by maintaining the extracellular space volume. This was evaluated using pentylenetetrazole- and pilocarpine-induced seizures caused due to the increased intracellular calcium and sodium concentration [9,10]. Moreover, the therapeutic value of chondroitin sulfate has been evaluated in various diseases like chronic bladder disease, antiaging, osteoarthritis, and inflammatory disorders [11,12,13,14]. Preclinical studies conducted by Zhang et al. [15] and Ju et al. [16] have shown the neuroprotective effect of chondroitin sulfate in Alzheimer’s and Parkinson’s diseases. As per the study conducted by Okamoto et al. [17], chondroitin sulfate has shown its anti-apoptotic and neuroprotective effects in rat brains by delaying neuronal cell death induced by excitatory amino acids in cultured neurons of the rat [15,16,17]. Many in-vivo and in-vitro studies have elicited the neuroprotective role of chondroitin and glucosamine amino acids in neurodegenerative diseases by reducing the levels of NF-kB and pro-inflammatory mediators like TNF-α, IL-1, and IL-6 causing neuronal death [18,19,20,21]. In addition, chondroitin sulfate possesses a neuroprotective effect by attenuating neuronal excitotoxic death and suppressing the caspase-3 activation [18]. The neuroprotective effect of chondroitin sulfate is due to its ready penetration across the blood-brain barrier as the glucosamine binds to the CD44 adhesion molecule receptor that gets elevated under the pathological condition of epileptogenesis [22]. Therefore, chondroitin sulfate can easily penetrate the blood-brain barrier by binding to the CD44 receptor, which is highly expressed under epileptic conditions, indicating the rupture of the blood-brain barrier and inflammation in the brain [23]. Additionally, perineuronal nets (PNNs) are also formed by chondroitin sulfate proteoglycans (CSPGs) that have a neuroprotective action against oxidative stress, which is potentially involved in neurodegeneration [22].

The purpose of this study was to evaluate the role of chondroitin sulfate in an antiepileptic agent. Therefore, the current research emphasized evaluating chondroitin sulfate in attenuating the kindling seizures threshold induced by pentylenetetrazole (PTZ) for generalized tonic or clonic seizures and the spontaneous seizures threshold induced by pilocarpine for status epileptics in mice. The current research work increases the understanding of the ECM changes and neuronal interaction alterations following an increase in the seizure activity in the brain, revealing the novel mechanistic role of chondroitin sulfate proteoglycan in epilepsy, and the obtained results provide an opportunity for the development of a new therapeutic approach in attenuating and preventing seizures.

## 2. Material and Method

### 2.1. Animals

Each group comprised six male Swiss albino mice weighing 25 ± 2 g (Ashirwad Pvt. Ltd., Chandigarh, India), which were employed for the present study and which were ethically approved by the institutional animal ethics committee and carried as per the guidelines of the Committee for Control and Supervision of Experiments on Animals (CPCSEA), Ministry of Environment and Forest, Government of India. Before starting the experimental protocol, the animals were provided free access to water and adequately housed in a condition of a 12 h cycle of light and dark.

### 2.2. Drugs and Chemicals

The chemicals and drugs used in the present study were purchased from a standard Indian supplier: M/s Sigma, St. Louis, MO, USA, company. The chemical pentylenetetrazole (M/s Sigma, St. Louis, MO, USA) and pilocarpine nitrate (FDC Limited, Aurangabad, India) were used for inducing disease in animals. Other treatment compounds, chondroitin sulfate (CS), standard valporate/sodium valproate (Yarrow Chem), and hyoscine butyl bromide (German Remedies Ltd., Mumbai, India), were employed and freshly prepared using a standard saline solution.

### 2.3. Anti-Convulsant Activity

A. Induction of the Pentylenetetrazole Kindled Model of Epilepsy in Mice

A pentylenetetrazole (PTZ)-induced kindling model representing partial and secondary generalized seizures was used to evaluate the antiepileptic effect of the treatment drug chondroitin sulfate. The sub-convulsive dose of pentylenetetrazole (40 mg/kg, i.p.) was administered on every alternative day for a total period of 15 days of study, and the severity of kindled seizures was assessed in terms of a composite kindled seizure severity score (KSSS) [24]. Before the administration of pentylenetetrazole, the treatment compound chondroitin sulfate at a dose of 100 & 200 mg/kg (p.o.) and valporate (110 mg/kg; i.p.) was administered at least 45 min before PTZ. The treatment with chondroitin sulfate was administered once daily, which was continuous throughout the experimental protocol of 15 days, i.e., starting from day 1 to day 15. KSSS quantification was assessed daily for 20 min in a plexiglass chamber after the induction of kindled seizures. The effect of treatment chondroitin sulfate against PTZ-induced kindled seizures was assessed on alternative days until day 15.

B. Induction of the Status Epilepticus Provoked Spontaneous Recurrent Seizure Activity in Mice

The pilocarpine model was used representing status epilepticus seizures; the sub-convulsive dose of pilocarpine (100 mg/kg, i.p) was administered every 20 min until the onset of spontaneous recurrent seizures started; and the seizure threshold was measured as per the spontaneous recurrent seizure severity score (SRSSS) for 20 min. The status epilepticus seizures were terminated after 40 min by using diazepam (3 mg/kg; i.p.) with normal saline. After 6 days of the induction of spontaneous recurrent seizures, the pilocarpine was administered at a single dose once daily; the treatment with chondroitin sulfate (100 & 200 mg/kg; p.o.) and valproate (110 mg/kg; i.p.) was administered 45 min to the application of pilocarpine-induced recurrent seizures. The treatment was started after 6 days. The behavioral parameters were set as per the spontaneous recurrent seizure severity score and were assessed every third day until day 37 [24]. Before administering pilocarpine, the hyoscine butyl bromide (1 mg/kg; i.p.) was given to avoid the peripheral cholinergic side effects.

### 2.4. Estimation of Oxidative Stress

For biochemical estimation, the animals were sacrificed by cervical dislocation. The brains were removed and homogenized in phosphate buffer (pH 7.4, 10% *w*/*v*) using a homogenizer for estimation of various biochemical parameters. The thiobarbituric acid, reduced glutathione, catalase, and superoxide dismutase (SOD) were estimated [25,26,27,28].

### 2.5. Estimation of Inflammatory Mediators

The quantification of caspase-3, TNF-α, NF-κB, IL-6, and IL-1β in the brain was done with the help and instructions provided by the Krishgen Biosystems Elisa kit, India.

### 2.6. Histopathological Evaluation of Brain Tissue

After completing the above behavior tests, mice were sacrificed, and their brains were transcardially perfused with 4% paraformaldehyde in a phosphate buffer solution with pH 7.4 for hematoxylin. Eosin staining histopathological evaluation for lesions of brain tissues under a light microscope was performed (Olympus microscope, Center Valley, PA, USA).

### 2.7. Experimental Protocol

In the present study, 10 groups were employed, and each group was comprised of 6 animals.

**Figure 1:**
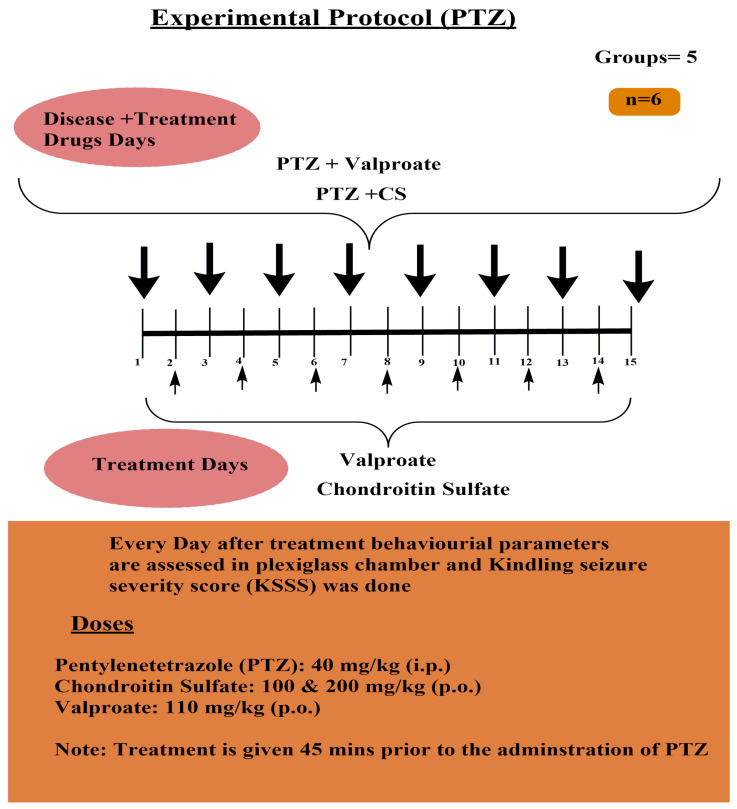
Experimental protocol for pentylenetetrazole-induced kindled seizure severity score assessment protocol.

A. Pentylenetetrazole-induced Kindled Seizure Severity Score Assessment Protocol (Figure 1)

**Figure 2:**
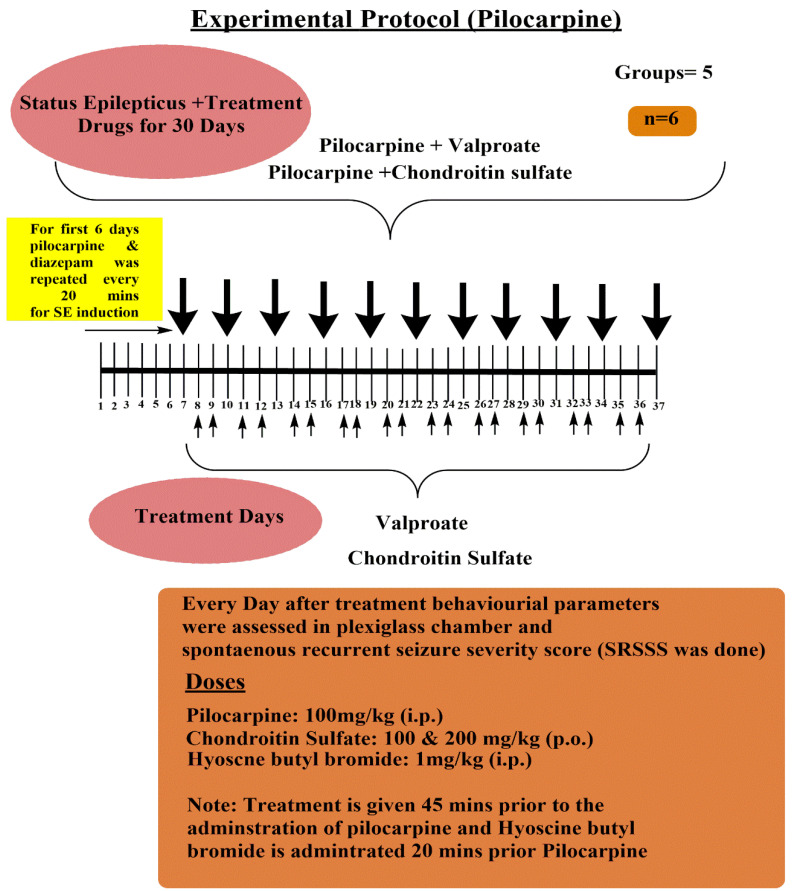
Experimental protocol for pilocarpine-induced spontaneous recurrent seizure severity score assessment protocol.

B. Pilocarpine-induced Spontaneous Recurrent Seizure Severity Score Assessment Protocol (Figure 2):

### 2.8. Molecular Docking Studies

To understand the binding interactions of chondroitin sulfate at the active pockets of TNF-alpha, IL-1β, and IL-6, the molecular docking simulation was carried out with the CHARMmbased docking tool and CDOCKER of Discovery Studio Client v20.1.0.19295 software [29,30]. The test compound was sketched and cleaned in Discovery Studio Client v20.1.0.19295 workspace, followed by energy minimization in the “Prepare Ligands” program of Discovery Studio Client at pH 7.4. The X-ray crystallographic structures of TNF-alpha (PDB ID: 2AZ5), IL-1β (PDB ID:1ITB) and IL-6 (PDB ID: 1ALU) were obtained from the Protein Data Bank (http://www.rcsb.org/pdb, accessed on 10 October 2021) and optimized for docking analysis [31]. The optimization protocol included adding hydrogen atoms, deleting water molecules, completing bond orders, and assigning hydrogen bonds. The test compound was docked into the protein active site using the CHARMmbased docking tool of the CDOCKER program. The binding energy of the hits with proteins was estimated as negative of the CDOCKER interaction energy [32].

### 2.9. Statistical Analysis

Data obtained from the study were statistically analyzed using one-way ANOVA followed by Tukey’s multiple range tests as post-hoc analysis. The results were shown as mean ± standard error of the mean (SEM). A value of *p* < 0.05 was considered to be statistically significant. The statistical analysis was performed using the Sigma Stat 6.0 software.

## 3. Results

### 3.1. Effect of Chondroitin Sulfate on PTZ-Induced Alteration in Kindling Severity Score in Mice

**Figure 3:**
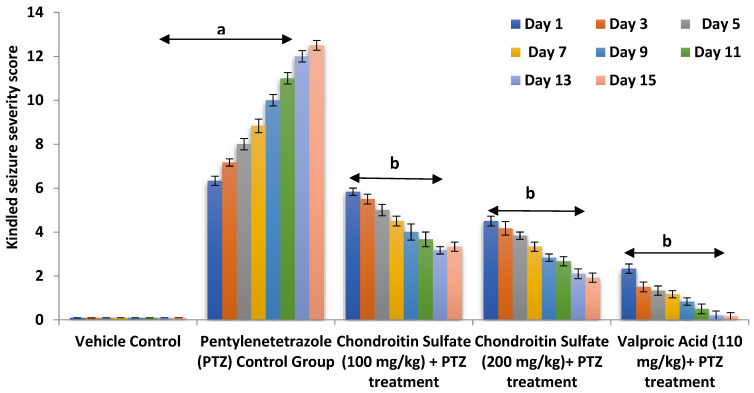
Antiepileptic effect of CS on PTZ-induced alteration in kindling severity score in mice. Values are expressed as mean ± SEM; ^a^ *p* < 0.01 vs. vehicle control; ^b^ *p* < 0.05 vs. PTZ control.

The concept of chondroitin sulfate in balancing the extracellular and intracellular ionic concentration was explored, as it provides a negative domain to the extracellular matrix and regulates the extracellular positive ion concentration. Therefore, to evaluate the therapeutic effect of chondroitin sulfate in the epileptic brain and supporting the extracellular space volume, the current study employed the pentylenetetrazole (PTZ) model. PTZ administration at a sub-convulsive dose of 40 mg/kg (i.p.) on alternative days for 15 days of study precipitated complex partial and secondarily generalized seizures in mice as reflected by a statistically significant increase (*p* < 0.01) in the composite kindling seizure severity scoring measured in terms of a concomitant rise in the severity of tonic-clonic, Straub tail, hunched back, stupor phase, wild running, and jumping seizures when compared to that of the vehicle-treated control groups. Co-administration of chondroitin sulfate (100 and 200 mg/kg; p.o.) significantly (*p* < 0.05) and dose-dependently, along with the reference drug valproate (110 mg kg^−1^; i.p.), attenuated complex partial and secondarily generalized seizures in mice, when measured in terms of the kindling severity scoring in mice accompanied by a marked decrease in the severity of the observed behavioral criteria, viz., tonic-clonic, Straub tail, hunched back, stupor phase, wild running, and jumping seizures, suggesting the role of restraining the influx of calcium and sodium ion preventing the depolarization of the membrane and possessing an anti-epileptic effect (Figure 3).

### 3.2. Effect of Chondroitin Sulfate on Pilocarpine-Induced Alteration in Spontaneous Recurrent Severity Score Mice

**Figure 4:**
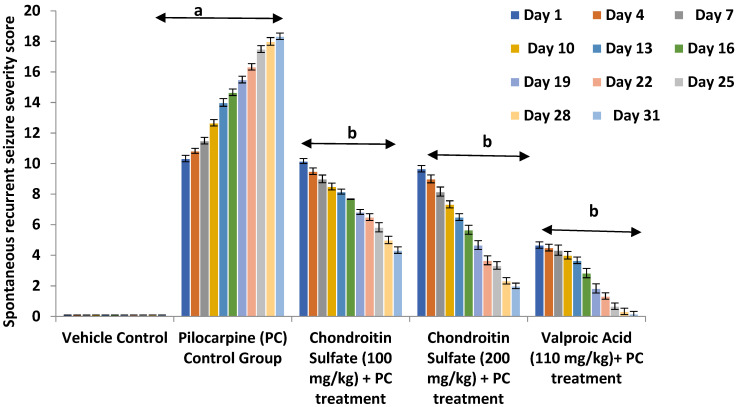
Antiepileptic effect of CS on pilocarpine-induced alteration in spontaneous recurrent severity score mice. Values are expressed as mean ± SEM; ^a^ *p* < 0.01 vs. vehicle control; ^b^ *p* < 0.05 vs. PC control.

To further investigate the effect of chondroitin sulfate as an anticonvulsant in status epilepticus by maintaining the intracellular influx of calcium ions and regulating the extracellular space volume, we established another pilocarpine model resembling status epilepticus by increased intracellular calcium-mediated spontaneous recurrent seizures. After induction of SE in mice, a single dose of pilocarpine was administered at a sub-convulsive dose of 100 mg/kg (i.p.) for every third day, which precipitated status epilepticus in mice as reflected by a statistically significant increase (*p* < 0.01) in the composite spontaneous recurrent seizures severity scoring measured in terms of a concomitant rise in the severity of wet dog shake, hyperlocomotion, and stereotypies (e.g., excessive grooming, rearing, and head movements); tonic-clonic; Straub tail, hunched back; stupor phase; wild running; and jumping seizures, when compared to that of the vehicle-treated control groups, for a study observation period of 31 days. Co-administration of chondroitin sulfate (100 & 200 mg/kg; p.o.) significantly (*p* < 0.05 each) and dose-dependently, along with the reference drug valproate (110 mg kg^−1^; i.p.), attenuated status epilepticus seizures in mice, when measured in terms of the spontaneous recurrent seizures severity scoring in mice accompanied by a marked decrease in the severity of the observed behavioral criteria, viz., wet dog shake, hyperlocomotion, and stereotypies (e.g., excessive grooming, rearing, and head movements); tonic-clonic, Straub tail; hunched back; stupor phase; wild running; and jumping seizures. Therefore, the result suggested the anti-epileptic effect of chondroitin sulfate in status epilepticus by retaining the extracellular calcium ion and decreasing the intracellular calcium ion concentration, which positively prevents the spontaneous recurrent seizures induced by pilocarpine (Figure 4).

### 3.3. Effect of Chondroitin Sulfate on Pentylenetetrazole Mediated Oxidative Stress in Mice

The antioxidant effect of chondroitin sulfate in epilepsy has been identified by studying the various biochemical oxidative stress parameters indicating the prolonged seizure-induced oxidative stress in epilepsy in terms of the levels of TBARS (a byproduct of lipid peroxidation that gets elevated under particular pathology indicating the brain oxidative stress tissue injury due to prolonged repetitive seizures), catalase, glutathione, and superoxide dismutase (SOD), representing brain tissue injury. Such a high sensitivity of the epileptic brain to oxidative stress during prolonged kindling seizures was induced by using pentylenetetrazole. The administration of PTZ at a subconvulsive dose of 40 mg/kg (i.p.) for 15 days, on alternative days, precipitated status epilepticus in mice as reflected by a statistically significant increase (*p* < 0.01) in the increased level of TBARS and decreased levels of catalase, glutathione, and superoxide dismutase (SOD), representing the oxidative stress in mice when compared to that of the vehicle-treated control groups. Co-administration of chondroitin sulfate (100 & 200 mg/kg; p.o.) significantly (*p* < 0.05) and dose-dependently, along with the reference drug valproate (110 mg kg^−1;^ i.p.), (*p* < 0.05) attenuated the levels of TBARS and increased the levels of catalase, glutathione, and superoxide dismutase (SOD) in mice. Therefore, this indicated the therapeutic effect of chondroitin sulfate preventing the prolonged seizure-induced oxidative stress in epilepsy (Table 1).

### 3.4. Effect of Chondroitin Sulfate on Pilocarpine-Mediated Oxidative Stress in Mice

To investigate the status of epilepticus-induced oxidative stress, the early cellular events of brain tissue injury with altered levels of biochemicals like antioxidants and increased lipid peroxidation were studied by using pilocarpine-induced spontaneous seizure-mediated oxidative stress. For a study observation period of 31 days, pilocarpine was administered at a sub-convulsive dose of 100 mg/kg (i.p.) for every third day, which precipitated status epilepticus in mice as reflected by a statistically significant increase (*p* < 0.01) in the increased level of TBARS and decreased levels of catalase, glutathione, and superoxide dismutase (SOD), representing the oxidative stress in mice when compared to that of the vehicle-treated control groups. Co-administration of chondroitin sulfate (100 & 200 mg/kg; p.o.) significantly (*p* < 0.01 each) and dose-dependently, along with the reference drug valproate (110 mg/kg^;^ i.p.), attenuated the levels of TBARS and increased levels of catalase, glutathione, and superoxide dismutase (SOD) in mice. Therefore, the present results suggested chondroitin sulfate in preventing the status epilepticus by reducing the oxidative stress causing neuronal damage (Table 2).

### 3.5. Effect of Chondroitin Sulfate on IL-1β, IL-6, NF-kB, and TNF-α Concentration in PTZ-Induced Kindling Seizures in Mice

For evaluating the anti-inflammatory potential of chondroitin sulfate, the levels of IL-1β, IL-6, NF-kB, and TNF-α were measured in pentylenetetrazole-induced excitotoxicity. The activation of the neuroimmune system with excessive migration of glial cells represented an increased expression of NF-kB-transcribing pro-inflammatory mediators. The study employed a pentylenetetrazole model by administration of PTZ at a sub-convulsive dose of 40 mg/kg (i.p.) for 15 days, which enhanced the levels of pro-inflammatory mediators (IL-1β, IL-6, and TNF-α) in mice as reflected by a statistically significant increase (*p* < 0.01) when compared with the vehicle control group. Co-administration of chondroitin sulfate (100 & 200 mg/kg; p.o.) significantly (*p* < 0.05) and dose-dependently, along with the reference drug valproate (110 mg kg^−1^; i.p.), (*p* < 0.05) attenuated the levels of IL-1β, IL-6, NF-kB, and TNF-α, representing a decrease in neuroinflammation, when compared with the PTZ control group (Table 3). The results provided evidence of the anti-inflammatory property of chondroitin sulfate in reducing the NF-kB expression and suppressing the neurotoxin pro-inflammatory (IL-1β, IL-6, and TNF-α) levels. Thus, chondroitin sulfate modulates glial cells and potentially regulates neuroinflammation-mediated excitotoxicity in epilepsy.

### 3.6. Effect of Chondroitin Sulfate on IL-1β, IL-6, NF-kB, TNF-α, and Caspase-3 Concentration in Pilocarpine-Induced Spontaneous Seizures in Mice

Status epilepticus seizures’ widespread brain inflammation suggest that the neuroinflammatory processes play a vital role in the occurrence and development of status epilepticus. Therefore, chondroitin sulfate possessing anti-inflammatory properties was evaluated against pilocarpine-induced spontaneous seizures associated with neuronal injury and perpetuating chronic inflammatory processes of migration of glial cells with increased NF-kB expression and elevated levels of neurotoxin pro-inflammatory mediators (IL-1β, IL-6, and TNF-α). Therefore, after induction of SE in mice, the study used the pilocarpine at a sub-convulsive dose of 100 mg/kg (i.p.) for 31 days, which precipitated the increased levels of pro-inflammatory mediators (IL-1β, IL-6, NF-kB, and TNF-α) in mice as reflected by a statistically significant increase (*p* < 0.01) as compared to the vehicle control group. Co-administration of chondroitin sulfate (100 & 200 mg/kg; p.o.) significantly (*p* < 0.05 each) and dose-dependently, along with reference drug valproate (110 mg kg^−1;^ i.p.), (*p* < 0.05) attenuated the levels of IL-1β, NF-kB, and TNF-α, when compared with the pilocarpine control group, representing a decrease in neuroinflammation (Table 4). Therefore, the above results suggested the potential role of chondroitin sulfate in reducing brain inflammation-mediated neuronal loss and recurrent seizures perpetuating chronic inflammation.

### 3.7. Effect of Chondroitin Sulfate on PTZ-Ediated Caspase-3 Level in Mice

The anti-apoptotic property of chondroitin sulfate was evaluated by determining the levels of caspase-3 in pentylenetetrazole-induced kindling seizures-mediated neuronal death. The excessive prolonged epileptic seizures cause mitochondrial oxidative stress-mediated neuronal death further marked by activated levels of caspase-3 enzyme-mediated apoptosis. Therefore, the administration of PTZ at a sub-convulsive dose of 40 mg/kg (i.p.) for 15 days increases the levels of the caspase-3 enzyme in mice as reflected by a statistically significant increase (*p* < 0.01), when compared with the vehicle control group. Co-administration of chondroitin sulfate (100 & 200 mg/kg; p.o.) significantly (*p* < 0.05 each) and dose-dependently, along with the reference drug valproate (110 mg kg^−1^; (i.p.)), attenuated the levels of caspase-3 when compared with the PTZ control group, representing the neuroprotective effect of chondroitin sulfate in epilepsy (Table 3). The above results indicate the anti-apoptotic effect of chondroitin sulfate in inhibiting the caspase-3 mediated apoptotic pathway and preventing the chronic seizure-induced neuronal death in epilepsy.

### 3.8. Effect of Chondroitin Sulfate on the Pilocarpine-Mediated Caspase-3 Level in Mice

The neuroprotective effect of chondroitin sulfate in status epilepticus was evaluated using the pilocarpine-model-mediated apoptotic neuronal death by activating caspase-3. After induction of SE in mice, pilocarpine was administered at a sub-convulsive dose of 100 mg/kg (i.p.) for 31 days, which precipitated the increased levels of caspase 3 in mice as reflected by a statistically significant increase (*p* < 0.01) as compared to the vehicle control group. Co-administration of chondroitin sulfate (100 & 200 mg/kg; p.o.) significantly (*p* < 0.05 each) and dose-dependently, along with the reference drug valproate (110 mg/kg; i.p.), attenuated the levels of caspase-3 when compared with the PC control group, representing the neuroprotective effect (Table 4). Therefore, the above results provided evidence of the anti-apoptotic effect of chondroitin sulfate in status epilepticus seizure-induced excitotoxic damage by activating the caspase-3 apoptotic pathway.

### 3.9. Effect of Chondroitin Sulfate on PTZ- and Pilocarpine-Mediated Histopathological Changes in Mice Brains

**Figure 5:**
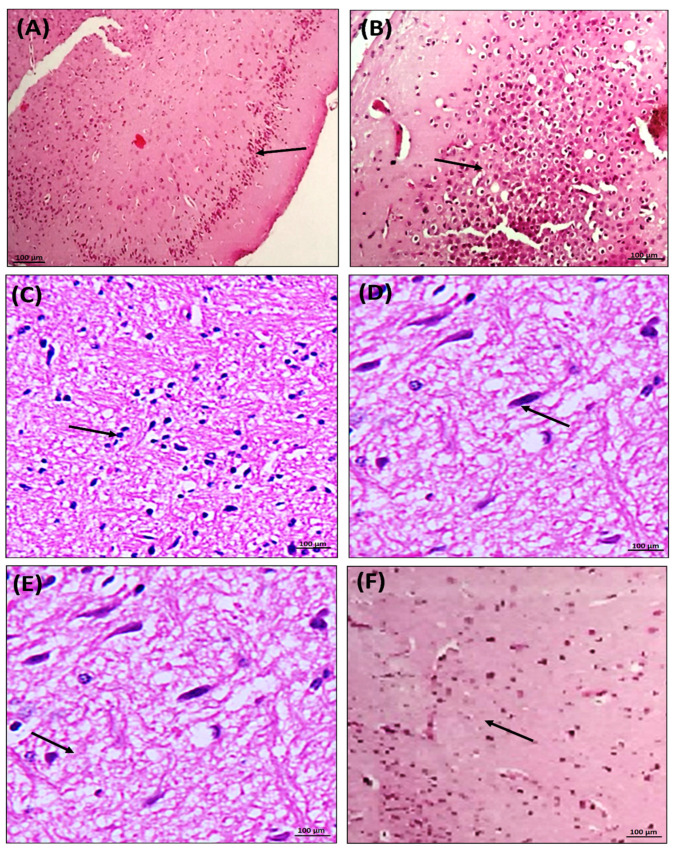
Antiepileptic effect of chondroitin sulfate on histomorphological changes marked by arrows against PTZ- and pilocarpine-induced seizures in mice. Scale bar of 100 µm. (**A**) is a normal histology of the vehicle control group with intact and appropriately sized nuclei and neural cells. In the pilocarpine control group, there was a distortion of neurons and neuronal condensation, necrosis, and pyknotic nuclei in pilocarpine-administered mice compared to the negative control mice represented in Figure (**B**) compared with the vehicle control group. Figures (**E**,**F**) with the treatment of chondroitin sulfate (CS) and valproic acid against pilocarpine-induced neuronal damages were recovered, evidenced by retaining the normal histology with decreased distortion and death compared to the pilocarpine control group. Figure (**D**) represents the pentylenetetrazole control group, which revealed distortion of neurons, neuronal condensation, and necrosis in PTZ-administered mice compared to the vehicle control group. Figure (**C**) with the treatment of chondroitin sulfate (CS) and valproic acid against PTZ-induced neuronal damages were recovered, which is evidenced by retaining the normal histology with decreased distortion and death compared to the pentylenetetrazole control group.

Evidence from histopathology accumulated the neuroprotective effect of chondroitin sulfate in epilepsy, suggesting the prevention of hippocampal neuronal damages that were analyzed after the administration of PTZ at a sub-convulsive dose of 40 mg/kg (i.p.) for 15 days, which precipitated the histopathological changes such as nuclear chromatin clumping, condensed cytoplasm, and fragmentation of the cells in mice brains. Co-administration of chondroitin sulfate groups (100 & 200 mg/kg; p.o.) and the reference drug valproate group (110 mg/kg; i.p.) prevented degenerative changes in the brain, which showed the neuroprotective effect of PTZ in treated mice. Thus, this indicated the neuroprotective effect of chondroitin sulfate against seizure-induced excitotoxic damage. The study provided evidence of the neuroprotective effect of chondroitin sulfate against neuronal loss associated with status epilepticus recurrent seizure, which was analyzed by the histopathological results. After induction of SE in mice, pilocarpine was administered at a sub-convulsive dose of 100 mg/kg (i.p.) for 31 days, which precipitated histopathological changes such as nuclear pyknosis and shrinkage of neuronal cells in mice brains. Co-administration of chondroitin sulfate (100 & 200 mg/kg; p.o.) and the reference drug valproate (110 mg/kg; i.p.) prevented degenerative changes in the neuronal tissue, which showed the neuroprotective effect in PC-treated mice. Therefore, the above results indicated chondroitin sulfate as an effective strategy in preventing such hippocampus neuronal degenerative changes in status epilepticus (Figure 5).

### 3.10. Molecular Docking Studies

**Figure 6:**
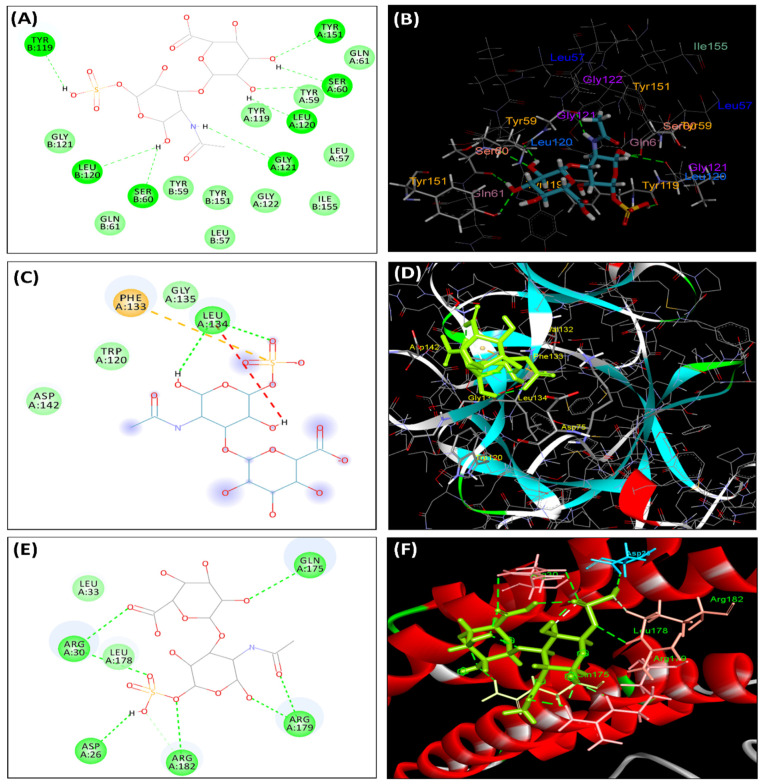
Interactions of chondroitin sulfate with active site of TNF-alpha pocket (2AZ5): (**A**) 2D interactions; (**B**) 3D interactions. Interactions of chondroitin sulfate with active site of IL-1β (1ITB) pocket: (**C**) 2D interactions; (**D**) 3D interactions. Interactions of chondroitin sulfate with active site of IL-6 (1ALU) pocket: (**E**) 2D interactions; (**F**) 3D interactions. The hydrogen bonds are indicated with green dashed lines, and the van der Waals forces are in white.

In order to discover the interaction patterns of chondroitin sulfate with TNF-alpha, IL-1β, and IL-6, docking studies were carried out using PDB IDs 2AZ5, 1ITB, and 1ALU crystal structures, respectively. Chondroitin sulfate at the active pocket of TNF-alpha, IL-1β, and IL-6 forms hydrogen bonds with additional van der Waals interactions with the crucial amino acid residues (Table 5, Figure 6).

## 4. Discussion

In the present investigation, administration of chondroitin sulfate concluded a significant (*p* < 0.05) and dose-dependent attenuation of PTZ- and pilocarpine-induced seizures in mice. Therefore, our data provided the evidence of pharmacological modulation of inflammatory mediators and antioxidant elements under chronic seizures, which have been suggested to provide neuroprotective effects during epileptic seizures by attenuating the kindling severity score induced by PTZ. Pilocarpine-induced spontaneous recurrent seizures were reduced in Swiss albino mice rodents. Nevertheless, further bimolecular studies are required to elucidate the involvement of chondroitin sulfate in epileptic mice.

The present study proposed a potential role of chondroitin sulfate in maintaining the electrical activity as an anticonvulsant, antioxidant, and anti-inflammatory molecule preventing neuronal death in epilepsy. Seizure-induced neuronal death is associated with alterations in neurotrophin-dependent pathways, excitotoxicity, apoptotic pathways, the inflammatory cascade, and oxidative stress [33,34,35]. In epilepsy, the brain dependency on oxygen consumption makes it more vulnerable to oxidative injury, which is exhibited by elevated oxygen free radicals [36]. In addition, the excessive repetitive seizures inducement of oxidative brain tissue injury is marked by shrinkage of neuronal tissue, causing a decrease in the extracellular space leading to the shift of ionic concentration between the intracellular and extracellular region, i.e., by increasing the intracellular space of ionic concentration as compared to the extracellular region of the neuronal cell [4,5,6]. The decreased extracellular space (ECS) increases the emphatic interactions between excitatory neurotransmitter signaling, leading to neuronal excitability [7]. The subsequent unreported role of the chondroitin sulfate (proteoglycan) component of the extracellular matrix in epilepsy encouraged the present research to propose the potential role of chondroitin sulfate in maintaining the electrical activity and in possessing a neuroprotective effect in epilepsy. Therefore, the present study provided preclinical evidence-based data on mice, depicting the reduction in epileptic seizures threshold when given orally. Chondroitin sulfate (proteoglycan) is widely distributed in the cartilage and tendons of animals and humans and is used by large-scale industries in manufacturing veterinary supplements, pet food products, or fertilizers. Various research studies have evaluated the potential therapeutic value of chondroitin sulfate as an anti-apoptotic and anti-inflammatory effect in certain diseased conditions like chronic bladder disease and osteoarthritis [11,12,13,14].

The present study demonstrated the natural cure for epilepsy by using a concentrated chondroitin sulfate dose-dependently (100 & 200 mg/kg; (p.o.)) against seizures induced by PTZ and pilocarpine treatments. In the current study, the sub-convulsive dose of 40 mg/kg (i.p.) of PTZ elicited a marked development in generalized seizures as assessed in terms of tonic-clonic seizures. From the above findings, the onset of seizures and the severity of tonic-clonic seizures were reduced significantly (*p* < 0.05) and dose-dependently in mice treated with CS (100 & 200 mg/kg; (p.o.)), when compared with the PTZ- and pilocarpine-treated groups, which was in line with previously published studies [34,35,36].

The results concluded the anti-epileptic effect of chondroitin sulfate. As mentioned in the literature, chondroitin sulfate (proteoglycan) is a component of the extracellular matrix that provides structural support to neurons. The extracellular matrix consists of fluid components and ions necessary for normal neuronal physiological function [2,16]. Furthermore, the literature supports the current research in providing a hypothetical idea that chondroitin sulfate provides a negative domain to the extracellular matrix with a high affinity of binding to cations extracellularly, further maintaining the ionic shift between extracellular and intracellular neuronal cells [2,16,37]. Therefore, the decreased severity of kindling seizures induced by pentylenetetrazole and pilocarpine in spontaneous recurrent seizures confirms the potential role of chondroitin sulfate in restraining the influx of intracellular positive ions such as calcium, sodium, and potassium, thereby preventing the depolarization of the membrane potential. An increase in extracellular potassium ions induces an increase in the resting potential of a neuron [16]. In this way, the above results indicate that chondroitin sulfate maintains extracellular space volume, which decreases due to PTZ- and pilocarpine-induced pathological brain structural changes of shrinkage of the brain, which are marked by a reduction in neuronal activity cell volume (condensed cytoplasm and DNA fragmentation) as evidenced from histopathology results. Thus, chondroitin sulfate maintains the extracellular space by regulating the ionic concentration in the extracellular space and reducing the synchronized frequent neuronal activity in the epileptic brain.

Additionally, chondroitin sulfate significantly (*p* < 0.05) and dose-dependently ameliorated PTZ- and pilocarpine-induced increased lipid peroxidation in animals marked by TBARS levels. Endogenous antioxidants (glutathione, SOD, and catalase) facilitate oxidative damage by neutralizing the effects of free radicals in the body [38]. In epilepsy, the excessive production of reactive oxygen free radicals (ROS) in seizures leads to alterations in endogenous antioxidant enzymes that result in oxidative damage to phospholipids with elevated levels of TBARS, which represent oxidative-stress-mediated neuronal damage in epilepsy [39]. In support of the above report, chondroitin sulfate tends to be an effective approach in epilepsy as our data revealed that subsequent treatment with chondroitin sulfate significantly (*p* < 0.05) and dose-dependently (100 & 200 mg/kg; p.o.) reinstated the endogenous antioxidant levels (glutathione, catalase, and superoxide dismutase (SOD)) and decreased the level of TBARS in brain tissue, indicating the reduction in oxidative stress as compared to the PTZ- and pilocarpine-induced diseased groups’ brain tissue. There was a decline in these antioxidants with an increase in the TBARS level, which represents the neuronal injury in the epileptic brain after the induction of PTZ- and pilocarpine-induced seizures in mice in line with the previous study [40]. Therefore, our results agree with previous studies of other pathological conditions that concluded that chondroitin sulfate exhibits free radical scavenging activity preventing the PTZ- and pilocarpine-induced chronic seizures’ mediated oxidative stress, further indicating that chondroitin sulfate possesses an anti-epileptic effect.

The neuroprotective effect of chondroitin sulfate was also explored in the current study using ELISA estimation of inflammatory mediators’ levels. The administration of chondroitin sulfate in mice significantly (*p* < 0.05) and dose-dependently, along with valproate (110 mg/kg, i.p.), decreased the levels of inflammatory mediators (interleukin-1 beta (IL-1β), interleukin-6 (IL-6), tumor necrosis factor-alpha (TNF-α), and NF-kB) as compared to the PTZ and pilocarpine disease control groups, indicating that the brain injury response of activated glial cells showed increased expression of NF-kB levels, which were also decreased in epileptic brain tissue with chondroitin-sulfate-treated animals. Further, the results of the current research are supported by various studies on epilepsy demonstrating the increased levels of pro-inflammatory mediators (IL-1 beta, IL-6, TNF alpha, and NF-kB) representing neuroinflammation in epileptic-brain-mediated generation of secondary seizures, which indicates the severity of seizures [41,42,43,44]. Thus, the results indicate the neuroprotective effect of chondroitin sulfate as an anti-inflammatory agent by suppressing the NF-kB and decreasing the inflammatory mediators (IL-1β, IL-6, and TNF-alpha) that act as biomarkers of brain inflammation. These mediators have been implicated in the pathogenesis of epilepsy by increasing the susceptibility of developing secondary seizures, which is consistent with earlier published data [16,18,44].

The various studies of immunochemical estimation in PTZ- and ilocarpine-induced seizures in mice have provided evidence of hippocampal neuronal and apoptotic death as confirmed by the TUNEL analyses, which confirmed that the mode of neuronal cell death resulted in identified fragmented double-stranded DNA, which was more significant of the hippocampus area of the brain that showed staining of apoptotic neuronal death in the hippocampus CA1 and CA3 regions that represent apoptotic neuronal death [45,46]. Therefore, the current study estimated the caspase-3 levels in the epileptic brain, and the elevated caspase-3 levels in the disease control groups was a marker of chronic seizure-induced neuronal injury in the hippocampus and entorhinal cortex-like brain areas in epilepsy. This result was justified by previous studies that confirmed increased levels of caspase-3, which indicate neuronal apoptotic death in the epileptic brain [45,46,47]. The current research provided evidence that treatment with chondroitin sulfate tends to be neuroprotective and that it significantly and dose-dependently (*p* < 0.05) decreases the caspase-3 levels in mice brain tissue, as compared to the PTZ- and pilocarpine-treated groups. This indicates that chondroitin sulfate has an anti-apoptotic effect that prevents neuronal death in epilepsy.

Further, the histopathological changes in the epileptic brain, including nuclear pyknosis, nuclear chromatin clumping, condensed cytoplasm, fragmentation of the cells representing damage in the brain by PTZ, and pilocarpine-induced seizures were estimated by using H&E staining [48,49,50]. The current study results confirmed the structural changes in PTZ- and pilocarpine-induced diseased brain samples with neuronal structure alterations compared to the vehicle control group that showed a normal texture and histology of neuronal tissue. The H&E staining of the treatment with chondroitin sulfate brain samples showed decreased brain structural changes in the neurons, such as nuclear chromatin clumping, condensed cytoplasm, and fragmentation of the cells, which were observed in PTZ-induced epileptic-seizure-mediated neuronal damage. The treatment with chondroitin sulfate showed a preventive effect in the pilocarpine-induced spontaneous recurrent seizures, causing brain damage like nuclear pyknosis and shrinkage of cells as compared to the disease groups.

The molecular docking study further confirmed the interaction pattern of chondroitin sulfate with different protein targets. The results concluded that chondroitin sulfate has a high interaction with inflammatory mediators (i.e., TNF-alpha, IL-1β, IL-6, and others that possess high binding energies). The position of chondroitin sulfate concerned the critical residues in the binding sites of TNF-alpha, IL-1β, and IL-6. The binding of chondroitin sulfate with some of the essential residues of these target sites may describe its anti-inflammatory mechanism of action.

Therefore, based on the above discussion, it may be suggested that chondroitin sulfate is a future target for epilepsy treatment. It could be further formulated as new dietary supplements, which may serve as a viable pharmacological therapeutic strategy in epilepsy. However, this needs to be preceded further and confirmed by immunohistochemical microarray and proteomic studies related to the evaluation of apoptotic mediators in neuronal cells in epilepsy to explore the mechanistic insight and proteins’ levels in-depth. These were limitations of this study.

## 5. Conclusions

With a growing interest and new dietary interventions in nutraceuticals, dietary supplements are considered beneficial therapy, meeting the demand of epileptic patients. Hence, the present findings indicate that chondroitin sulfate possesses a strong neuroprotective potential against seizures with the aim of improving the quality of life.

## Figures and Tables

**Table 1:** Antiepileptic effect of CS on biochemical estimation parameters in PTZ-induced kindling seizures. Values are expressed as mean ± S.D.; ^a^ *p* < 0.01 vs. vehicle control; ^b^ *p* < 0.05 vs. PTZ control.

Groups	TBARs (nmol/mg of Protein)	Catalase (µM of H_2_O_2_ Oxidised/min/mg Protein)	Glutathione (µM of Glutathione/mg of Protein)	Superoxide Dismutase (SOD) (U/mg of protein)
Vehicle control group	3.4 ± 0.99	39.73 ± 2.15	48.75 ± 2.76	35.8 ± 2.64
Pentylenetetrazole (PTZ) control	22.1 ± 2.32 ^a^	14.26 ± 1.19 ^a^	17.01 ± 3.10 ^a^	12.95 ± 0.99 ^a^
Chondroitin sulfate (100 mg/kg) + PTZ treatment	11.3 ± 1.57 ^b^	21.2 ± 1.75 ^b^	26.5 ±1.47 ^b^	20.93 ± 1.48 ^b^
Chondroitin sulfate (200 mg/kg) + PTZ treatment	6.11 ± 0.57 ^b^	34.73 ± 1.60 ^b^	41.3 ± 1.53 ^b^	30.23 ± 1.24 ^b^
Valproic acid (110 mg/kg)+ PTZ treatment	4.46 ± 0.52 ^b^	38.48 ± 2.09 ^b^	45.4 ± 3.93 ^b^	33.85 ± 3.46 ^b^

**Table 2:** Antiepileptic effect of CS on biochemical estimation parameters in pilocarpine induced-spontaneous recurrent seizures. Values are expressed as mean ± S.D.; ^a^ *p* < 0.01 vs. vehicle control; ^b^ *p* < 0.05 vs. PC control.

Groups	TBARs (nmol/mg of Protein)	Catalase (µM of H_2_O_2_ Oxidised/min/mg Protein)	Glutathione (µM of Glutathione/mg of Protein)	Superoxide Dismutase (SOD) (U/mg of Protein)
Vehicle control group	3.4 ± 0.99	39.73 ± 2.15	48.75 ± 2.15	35.8 ± 2.64
Pilocarpine control	24.76 ± 3.11 ^a^	11.36 ± 0.93 ^a^	15.38 ± 0.93 ^a^	10.86 ± 1.23 ^a^
Chondroitin sulfate (100 mg/kg) + pilocarpine treatment	12.3 ± 1.78 ^b^	20.96 ± 1.53 ^b^	25.06 ± 1.53 ^b^	15.6 ± 0.81 ^b^
Chondroitin sulfate (200 mg/kg) + pilocarpine treatment	6.38 ± 0.62 ^b^	34.06 ± 2.01 ^b^	39.96 ± 2.01 ^b^	29.56 ± 1.24 ^b^
Valproic acid (110 mg/kg) + pilocarpine treatment	4.81 ± 0.71 ^b^	38.15 ± 2.67 ^b^	44.36 ± 2.67 ^b^	32.9 ± 2.02 ^b^

**Table 3:** Neuroprotective effect of chondroitin sulfate on IL-1β, IL-6, NF-kB, and TNF-α concentration in PTZ-induced kindling seizures in mice. Values are expressed as mean ± S.D.; ^a^ *p* < 0.01 vs. vehicle control; ^b^ *p* < 0.05 vs. PTZ control.

Groups	IL-1β (pg/mg of Tissue)	IL-6 (pg/mg of Tissue)	NF-kB (pg/mg of Tissue)	TNF-α (pg/mg of Tissue)	Caspase-3 (pg/mg of Tissue)
Vehicle control group	47.25 ± 1.61	39.16 ± 2.37	36.83 ± 1.40	30.43 ± 1.19	0.71 ± 0.147
Pentylenetetrazole (PTZ) control	155.86 ± 3.79 ^a^	106.83 ± 2.99 ^a^	83.46 ± 3.19 ^a^	74.8 ± 2.38 ^a^	8.3 ± 0.43 ^a^
Chondroitin sulfate (100 mg/kg) + PTZ treatment	110.4 ± 4.59 ^b^	80.5 ± 3.83 ^b^	66.15 ± 2.53 ^b^	55.68 ± 3.24 ^b^	6.45 ± 0.92 ^b^
Chondroitin sulfate (200 mg/kg) + PTZ treatment	62.1 ± 3.87 ^b^	54.3 ± 2.59 ^b^	48.13 ± 2.18 ^b^	44.5 ± 2.75 ^b^	4.51 ± 0.46 ^b^
Valproic acid (110 mg/kg) + PTZ treatment	54.75 ± 2.16 ^b^	45.16 ± 1.45 ^b^	41.08 ± 1.58 ^b^	37.56 ± 1.35 ^b^	3.68 ± 0.33 ^b^

**Table 4:** Neuroprotective chondroitin sulfate on IL-1β, IL-6, NF-kB, TNF-α, and caspase-3 concentration in pilocarpine-induced spontaneous seizures in mice. Values are expressed as mean ± S.D.; ^a^ *p* < 0.01 vs. vehicle control; ^b^ *p* < 0.05 vs. PC control.

Groups	IL-1β (pg/mg of Tissue)	IL-6 (pg/mg of Tissue)	NF-kB (pg/mg of Tissue)	TNF-α (pg/mg of Tissue)	Caspase-3 (pg/mg of Tissue)
Vehicle control group	47.25 ± 1.61	39.61± 2.37	36.88 ± 1.40	30.43± 1.19	0.71 ± 0.14
Pilocarpine control	175.66 ± 4.36 ^a^	131.83± 4.73 ^a^	93.18± 1.92 ^a^	83.46± 3.83 ^a^	11.01 ± 0.90 ^a^
Chondroitin sulfate (100 mg/kg) + pilocarpine treatment	120.66 ± 3.66 ^b^	93.51 ± 3.11 ^b^	68.48 ± 2.57 ^b^	58.55 ± 4.02 ^b^	6.6 ± 0.49 ^b^
Chondroitin sulfate (200 mg/kg) + pilocarpine treatment	64.13 ± 3.99 ^b^	59.3 ± 2.79 ^b^	51.98 ± 2.45 ^b^	46.35 ± 3.52 ^b^	4.8 ± 0.52 ^b^
Valproic acid (110 mg/kg) + pilocarpine treatment	56.2 ± 3.41 ^b^	44.9 ± 2.56 ^b^	43.35 ± 1.96 ^b^	39.31 ± 1.20 ^b^	3.9 ± 0.25 ^b^

**Table 5:** Molecular docking of chondroitin sulfate on the active pocket of protein molecules.

Protein	Binding Interaction Energy	Type of Interaction	Bonding Amino Acids
TNF-alpha	+	H-bond	Tyr 151, Ser 60, Leu120, Gly121
		van der Waals	Tyr59, Leu57, Gln61, Tyr119, Gly122
IL-1β	−16.50	H-bond	Leu134, Phe133
		van der Waals	Gly135, Trp120, Asp142
IL-6	−18.68	H-bond	Gln175, Arg30, Asp26, Arg179, Arg182
		van der Waals	Leu33, Leu178

## Data Availability

All data generated or analyzed during this study are included in this article. There are no separate or additional files.

## Sample Availability

Samples of the compounds are not available from the authors.
